# The Relationship Between Parental Psychological Control and Non-Suicidal Self-Injury in Adolescents: A Chain Mediation Model

**DOI:** 10.3390/bs16010145

**Published:** 2026-01-20

**Authors:** Xiaoxi Jiang, Tong Yue, Sizhe Wang

**Affiliations:** 1 School of Psychology, Southwest University, Chongqing 400715, China; jiangzhuhan@163.com (X.J.); fx46922@163.com (S.W.); 2 Research Center for Psychology and Social Development, Southwest University, Chongqing 400715, China

**Keywords:** non-suicidal self-injury (NSSI), parental psychological control, resilience, self-disgust, adolescents

## Abstract

Background: Non-suicidal self-injury (NSSI) is a significant public health issue that threatens the physical and mental well-being of adolescents. Identifying associated factors is a crucial step toward effective intervention. Methods: This study analyzed data from 463 adolescents (42.12% boys; mean age = 16.21 years, range 12–18) to explore the relationships between NSSI, parental psychological control, self-disgust, and resilience. Results: Multivariate logistic regression indicated that parental psychological control, self-disgust, and resilience were significantly associated with NSSI. Furthermore, in the chain mediation model, self-disgust and resilience significantly mediated the relationship between parental psychological control and NSSI. Conclusions: This study examines the relationships between parental psychological control, self-disgust, resilience and NSSI in adolescents, providing theoretical insights and practical implications for developing intervention and prevention strategies.

## 1. Introduction

Non-Suicidal Self-Injury (NSSI) refers to deliberate, repeated injury of the body’s surface tissue (e.g., cutting, burning, and scratching) without suicidal intent (Nock et al., 2006; Jiang et al., 2011). This behavior, though not socially sanctioned and typically non-lethal or of low lethality, is highly dangerous and associated with various psychological disorders and suicide risks (Jiang et al., 2011; Sinclair et al., 2010). Research by Nock et al. (2006) indicates that 70% of adolescents who engage in NSSI have attempted suicide at least once, while 55% have made multiple suicide attempts.

According to the most recent edition, the Diagnostic and Statistical Manual of Mental Disorders, Fifth Edition, Text Revision (DSM-5-TR), NSSI is recognized as a condition that may be the focus of clinical attention. NSSI is prevalent among adolescent populations worldwide, with its incidence rate increasing annually (Wen et al., 2020). According to a survey conducted in Australia and six European countries, lifetime prevalence of self-harm was reported by 13.5% of girls and 4.3% of boys in the 14–17 age group (Carvalho et al., 2017). S. C. Zhang et al. (2016) surveyed Chinese middle school students and found that the prevalence of NSSI was 26.4% among girls and 28.6% among boys. Epidemiological studies further reveal that the incidence of NSSI among Chinese adolescents is higher than in Western countries (Jiang et al., 2011) and continues to rise (Wen et al., 2020).

Given the high prevalence of NSSI, identifying its risk factors is crucial. Research suggests that both early trauma and individual susceptibility play significant roles (Jiang et al., 2011). Among early traumatic experiences, abuse and neglect have received considerable attention. Early studies found that, based on self-reports, 79% of individuals who engaged in NSSI had experienced childhood abuse or neglect during childhood (Yates et al., 2008). During early development, children primarily interact with their parents within the family context, making parental upbringing crucial for healthy development. Negative parenting styles are often linked to experiences of abuse and neglect (Y. Wang & Li, 2012). Specifically, parental psychological control, defined as intrusive, manipulative practices (e.g., guilt induction and love withdrawal) that restrict a child’s psychological autonomy (Chen et al., 2023), has garnered increasing research attention due to its insidious and emotionally manipulative nature. It has been clearly associated with and established as a significant predictor of adolescent NSSI (Bureau et al., 2010; Qu et al., 2022). Therefore, elucidating the underlying psychological mechanisms through which parental psychological control contributes to NSSI is not only theoretically essential for advancing our understanding of developmental psychopathology but also critically informs the development of targeted prevention and intervention strategies.

### 1.1. Conceptual Framework: Self-Disgust and Resilience as Mediators

Building upon the established link between parental psychological control and NSSI, recent research has focused on identifying the specific psychological pathways that may explain this association. Self-disgust and psychological resilience have emerged as promising candidate mediators in this relationship.

#### 1.1.1. Self-Disgust as a Mediator

Self-disgust refers to persistent or recurrent experiences of revulsion toward aspects of oneself, accompanied by avoidance and rejection behaviors (Ille et al., 2014). During adolescence—a critical period for self-concept development, this self-disgust manifests with particular sensitivity and destructiveness. Theoretically, self-disgust can be conceptualized as a form of self-criticism comprising two core components of self-hatred and self-critical tendencies, where the cognitive aspect of self-criticism shows established links with NSSI (Smith et al., 2015). Joiner’s Interpersonal Theory of Suicide (ITS) argues that perceived burdensomeness (which includes self-disgust dimensions) and thwarted belongingness jointly contribute to suicidal ideation (Joiner et al., 2016; van Orden et al., 2010). When intense self-disgust generates suicidal ideation, but individuals face insufficient capability for suicide or death anxiety, NSSI may emerge as a substitute behavior. Furthermore, when externally directed, disgust becomes self-targeted and individuals develop profound rejection of their traits, behaviors, or existence. Such overwhelming self-disgust exceeds emotional tolerance thresholds, making NSSI an immediate—though maladaptive—emotion regulation strategy to temporarily alleviate this distress. Empirical support comes from Nock and Favazza (2009), whose study of 30 NSSI adolescents (aged 12–19) found that 48.5% reported self-directed anger and 42.7% exhibited self-hatred prior to self-injury, confirming self-disgust as a predominant pre-NSSI psychological state.

As an intrusive parenting style involving guilt induction and love withdrawal (Barber, 1996), parental psychological control constitutes a significant risk factor for adolescent self-disgust development. First, conditional acceptance binds children’s self-worth to compliance, fostering self-negation when parental expectations remain unmet (Soenens et al., 2010). Second, emotionally cold and rejection-laden environments hinder the formation of a positive self-concept. Such upbringing cultivates an automatic self-blaming attribution style—individuals habitually attribute negative events to inherent, unchangeable personal flaws (X. Li & Qian, 2000). Neuroimaging evidence corroborates this: adolescents experiencing high psychological control show reduced dorsolateral prefrontal cortex activation during maternal criticism processing (Lee et al., 2015) but heightened amygdala reactivity in social evaluation contexts (Guyer et al., 2015). This neural hypersensitivity to negative evaluation correlates strongly with self-criticism (Longe et al., 2010), which is the core component of self-disgust (Overton et al., 2008).

According to the extant literature, we hypothesize that the pathway from parental psychological control to adolescent NSSI risk involves amplified self-disgust. Specifically, among adolescents subjected to high psychological control, conditional acceptance and emotional deprivation intensify self-negation tendencies, thereby exacerbating self-disgust experiences that prompt NSSI as an emotion regulation strategy. This hypothesis elucidates the intrinsic emotional mechanism (i.e., the mediating pathway of self-disgust) underlying parental psychological control’s influence on NSSI, while highlighting the pivotal role of distorted self-concept in adolescent self-injury development.

#### 1.1.2. Resilience as a Mediator

Resilience, defined as the capacity for positive adaptation in adverse circumstances (Yuan et al., 2024), serves as a critical protective factor in adolescent emotional regulation and behavioral outcomes. It encompasses an individual’s ability to utilize intrinsic resilient traits to effectively cope with stressors and rapidly adapt to challenging situations (Yuan et al., 2024). Highly resilient individuals demonstrate superior emotion regulation capacities, preferentially employing adaptive strategies over somatic expressions to mitigate negative effects, thereby exhibiting reduced impulsive NSSI (Troy & Mauss, 2011). Empirical evidence supports this protective function: Gao et al. (2020) identified resilience as a significant negative predictor of NSSI among adolescents, while Zhong and Zhang’s (2024) study of depressive disorders confirmed inverse correlations between resilience scale dimensions and NSSI frequency.

The development of resilience, particularly during the formative adolescent period, is fundamentally contingent upon secure family environments. Studies indicate that excessive parental psychological control is associated with a disruption in this foundational sense of security through dual mechanisms: First, persistent emotional manipulation (e.g., guilt induction, love withdrawal) impedes autonomy development, compromising the acquisition of independent problem-solving competencies (Barber, 1996; Soenens et al., 2010). Second, conditional regard fosters maladaptive emotion regulation patterns, with negative responsiveness strongly predicting emotional dysregulation strategies (Rogers et al., 2021). Chinese longitudinal data also reveals that parental psychological control significantly attenuates adolescent resilience levels, indirectly elevating depression risk through this pathway (Q. Wang et al., 2020). Collectively, these findings demonstrate how psychological control undermines resilience development by constraining autonomy and distorting regulatory processes, rendering adolescents vulnerable to engagement in NSSI.

Building upon previous research, we hypothesize that resilience mediates the relationship between parental psychological control and adolescent NSSI. Specifically, elevated parental psychological control is predicted to inhibit resilience development, thereby increasing vulnerability to NSSI engagement. This hypothesis aims to elucidate the intrinsic psychological mechanism underlying parenting effects on self-injury while highlighting the buffering role of resistance against pathological outcomes.

#### 1.1.3. A Sequential Pathway from Self-Disgust to Resilience

Current research indicates that self-disgust, defined as an affective–cognitive experience marked by persistent self-denial and self-rejection (Ille et al., 2014), directly depletes the key internal resources necessary for psychological resilience. It significantly compromises people’s capability to navigate challenges and consequently inhibits the development of resilience. The internal resources of psychological resilience, which are crucial for maintaining adaptation or rapid recovery in the face of stress and challenges, include positive self-perception, emotion regulation skills, problem-solving abilities, and self-efficacy (H. Li & Zhang, 2006). The following elaborates on the mechanisms through which self-disgust erodes these internal resources from three key aspects.

First, self-disgust severely erodes positive self-perception. While resilient individuals possess positive self-schemas and a sense of environmental control (S. C. Zhang et al., 2016), self-disgust reinforces a negative cognitive state of self-unacceptance, inhibiting the development of adaptive coping beliefs and increasing susceptibility to helplessness under pressure. Second, psychological resilience correlates positively with the application of adaptive cognitive emotion regulation strategies (Tang & Zhou, 2014). High levels of self-disgust may impair emotion regulation, predisposing individuals to maladaptive strategies like suppression and avoidance (Smith et al., 2015), thereby exacerbating emotional dysregulation. Finally, self-disgust is likely to inhibit proactive coping, leading to avoidance rather than active problem-solving (Xi et al., 2012). Therefore, self-disgust compromises an individual’s capacity to cope with stress by undermining self-worth, disrupting emotional regulation, and inhibiting proactive behaviors. Consequently, self-disgust may elevate adolescents’ risk of NSSI by diminishing psychological resilience.

### 1.2. The Present Study and Hypotheses

**Figure 1:**
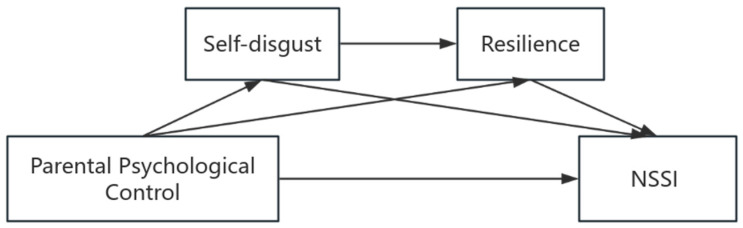
Conceptual model.

With evolving social attitudes and adjustments in fertility policies, contemporary Chinese adolescents are growing up in an environment characterized by increased family attention and intensified psychological pressure. Against this background, parental psychological control and individual psychological traits jointly influence their psychological adaptation. Therefore, this study aims to investigate the association between parental psychological control and adolescents’ NSSI by testing a chain mediation model that explores the potential sequential roles of self-disgust and resilience. This study helps to reveal the potential psychological mechanisms of NSSI and provides a valuable theoretical framework for understanding how the NSSI process might unfold. Guided by theory and existing evidence, Figure 1 presents a conceptual model to investigate the potential relationships among the variables. This study’s hypotheses were as follows:

**Hypothesis 1.**  *Parental psychological control will be a significant positive predictor of adolescents’ NSSI.*

**Hypothesis 2.**  *Parental psychological control contributes to NSSI by reducing resilience, which acts as a mediating factor.*

**Hypothesis 3.**  *Parental psychological control contributes to NSSI by increasing self-disgust, which acts as a mediating factor.*

**Hypothesis 4.**  *Parental psychological control predicts increased levels of self-disgust, which, in turn, is associated with reduced resilience, ultimately leading to a higher risk of engaging in NSSI.*

## 2. Materials and Methods

### 2.1. Participants

A total of 531 students from a middle school in Chongqing, China, were recruited using convenience sampling. All participants provided informed consent, which was obtained from either the participants themselves or their legal guardians. After excluding 68 participants who did not complete the surveys or failed the two attention checks, 463 participants (87.19%) remained, comprising 195 boys and 267 girls (one participant did not report gender information), aged 12 to 18 years (M = 16.21, SD = 1.45).

Among the participants, 40.6% (n = 188) were only children, while 59.0% (n = 273) were not only children; two participants did not fill in the information.

For the family residence, 81.2% (n = 376) of participants lived in the city and 18.1% (n = 84) of participants lived in the countryside; three participants did not fill in their residence information.

In terms of family types, 83.6% (n = 387) came from two-parent families, 9.1% (n = 42) came from single-parent families, 5.4% (n = 25) came from regrouped families and 1.1% (n = 5) came from other types of families; data on family type were missing for four participants.

### 2.2. Instruments and Measures

*Parental Control Scale (PCS).* Parental psychological control was assessed using the Chinese version of the PCS by Q. Wang et al. (2020). This 18-item instrument measures three dimensions: guilt induction, love withdrawal and authority assertion. Participants responded to items (e.g., “When my parents criticize me, they remind me of my previous mistakes”) on a 5-point Likert scale ranging from 1 (complete non-compliance) to 5 (complete compliance), with higher scores reflecting greater perceived psychological control. In this study, Cronbach’s alpha for parental psychological control was 0.95, and Cronbach’s α coefficients for the three dimensions of guilt induction, love withdrawal and authority assertion were 0.92, 0.88, and 0.87, respectively.

*Self-disgust Scale (SDS).* The Chinese adaptation (Xiong et al., 2019) of the Self-Disgust Scale (SDS; Overton et al., 2008) was employed to evaluate self-disgust. Of its 17 items, 11 items were related to the content and structure of the scale (e.g., “I find myself repulsive”), and the total score was calculated; the 6 items were neutral filler statements used to balance negative statements in the items and were not scored. Responses were recorded on a 7-point Likert scale, where 1 represents “strongly agree” and 7 represents “strongly disagree”. Cronbach’s alpha coefficient of the scale in this study was 0.78.

*Adolescent Non-suicidal Self-Injury Assessment Questionnaire (NSSI).* This study used the adolescent non-suicidal self-injury questionnaire developed by Wan et al. (2018) to measure the participants’ NSSI over the past 12 months. The scale includes 12 items and is rated on a 5-point scale from “no” to “always”. The items include 12 types of self-injury, such as intentional pinching, scratching, biting, hitting hard objects on one’s head, and pulling off one’s own hair. Referring to the definition of NSSI, “There were more than 5 instances of self-inflicted bodily injury without suicidal intent in the past 1 year” in DSM-5 and the operational definition of NSSI by scale developers, this study decided to classify participants with a total score of ≥5 into the “NSSI group”. Cronbach’s alpha coefficient of the scale in this study was 0.94.

*Resilience Scale.* This study used the resilience scale developed by Hu and Gan (2008), consisting of 27 items (e.g., “Failure always makes me feel discouraged”), scored on a 5-point Likert scale: 1 = completely disagree and 5 = completely agree. The scale includes five factors: goal concentration, emotional control, positive cognition, family support and interpersonal assistance. The reliability and validity of the scale have been well validated in the study of resilience among Chinese adolescents. Cronbach’s alpha coefficient of the scale in this study was 0.91.

### 2.3. Procedure

The data collection was conducted by two well-trained researchers using questionnaires, and participants completed them in class. To ensure compliance with ethical standards, ethical approval was obtained from the Ethics Committee of the University (Approval No. SWU-ECHR-20250224), and the procedures adhered to the principles of the Declaration of Helsinki. All participants and their legal guardians were fully informed about the study’s purpose, procedures, potential risks, and safeguards. Written informed consent was secured from either the adolescents or their guardians before participation. Additionally, participants were assured of the anonymity and confidentiality of their responses, which would be used solely for academic purposes.

### 2.4. Bias

Potential limitations include self-reporting bias from the questionnaire format, which could affect responses. This was mitigated by ensuring anonymity and stressing voluntary participation. Additionally, convenience sampling limits how broadly the results can be generalized beyond the sampled Chinese urban middle school students. Common method bias was evaluated using Harman’s single-factor test; the identification of 13 distinct factors (eigenvalues > 1) suggested its presence was not a primary concern. The highest factor accounted for 29.94% of the variance, which is below the critical threshold of 40%, suggesting that common method bias was not significant.

### 2.5. Statistical Methods

Statistical analyses were performed using SPSS 26.0, and the macro-program Process plugin of SPSS compiled by Hayes was used for multiple mediation analysis. The study removed subjects with more than 1 missing value, resulting in 463 subjects. For the remaining data, the multiple imputation method was used for 20 multiple imputations to generate 20 complete data sets. All analysis models were fitted separately on 20 data sets, and the final parameter estimates were combined according to Rubin’s law. First, to take full advantage of the variability of the data, Pearson correlation analyses were conducted to examine the bivariate relationships among all variables, treating NSSI scores as continuous. At the same time, the relevant analysis provided preliminary evidence and a basis for the subsequent model establishment. Second, in order to make the study more clinically relevant and facilitate understanding of the impact of variables on the risk of NSSI occurrence, binary logistic regression was employed, with NSSI status (presence/absence) as the dichotomous outcome variable, to assess the predictive utility of the factor set. Finally, significant predictors identified from the preceding analyses were incorporated into a serial mediation model, and Model 6 was used to examine the hypothesized indirect pathways.

## 3. Results

### 3.1. Descriptive Statistics

Among the total sample of 463 adolescents, 75 participants (16.2%) were classified into the NSSI group (total score ≥ 5), while the remaining participants (83.8%) comprised the non-NSSI group. For demographic variables, Chi-square tests revealed a significant difference between the two groups for gender, but no significant differences in grade, family type, or only-child status. Girls showed more NSSI behaviors than boys (*p* < 0.01).

### 3.2. Correlations for All Variables

Pearson correlation analysis showed that resilience was negatively correlated to the NSSI score, while parental psychological control and self-disgust were positively correlated to the NSSI score. See Table 1 for details. The statistical significance level was set at α = 0.05. Shown in the table are the combined estimates based on the multiple imputation model. As a sensitivity analysis, the results obtained by the mean-filling method were basically consistent in direction and significance.

### 3.3. Binary Logistic Regression

In order to test the independent association of parental psychological control, self-disgust, resilience and NSSI, hierarchical logistic regression analysis was used. First, an unadjusted model including parental psychological control, self-disgust, resilience, and NSSI was constructed (Model 1). The Nagelkerke R^2^ of the model is 0.44. The logistic regression coefficients indicate that higher levels of parental psychological control and self-disgust were associated with a greater likelihood of engaging in NSSI, whereas higher resilience was associated with a reduced likelihood. See Table 2 for details.

Subsequently, on the basis of Model 1, an adjusted model (Model 2) was constructed by simultaneously incorporating a set of prespecified potential confounders, including gender, grade, family type, and only-child status. The robustness of the association was assessed by comparing the changes in the effect sizes and their confidence intervals of parental psychological control, self-disgust, and resilience in the two models. The Nagelkerke R^2^ of the model is 0.47. See Table 3 for details.

The significant positive correlation between parental psychological control, self-disgust and NSSI, and the significant negative correlation between resilience and NSSI, remained robust after controlling for potential confounders. After incorporating all control variables simultaneously in Model 2, although the effect of parental psychological control slightly decreased, it still maintained a high level of statistical significance (*p* < 0.05).

### 3.4. Analysis of Multiple Mediating Effects

This study utilized Model 6 of PROCESS 4.0 to explore the mediating role of self-disgust and resilience in the relationship between parental psychological control and NSSI behavior, while controlling for grade, gender, only-child status and family type as covariates. As the NSSI is a dichotomous variable, the path analysis framework based on logistic regression was adopted. The bias-corrected percentile Bootstrap method was used with 5000 repeated samplings to estimate confidence intervals for indirect effects.

**Figure 2:**
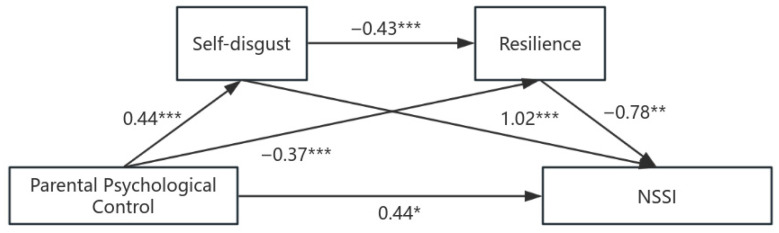
Chain mediation effect diagram of self-disgust and resilience. *Note.* Path coefficients are unstandardized regression coefficients (*B*). * *p* < 0.05; ** *p* < 0.01; *** *p* < 0.001.

As shown in Table 4, path analysis highlighted that parental psychological control significantly positively predicted self-disgust (*B* = 0.44, *p* < 0.001); self-disgust significantly negatively predicted resilience (*B* = −0.43, *p* < 0.001); and a decrease in resilience significantly increased the risk of NSSI (*B* = −0.78, *p* < 0.001, OR = 0.46). Parental psychological control significantly negatively predicted resilience (*B* = −0.37, *p* < 0.001), and self-disgust significantly positively predicted NSSI (*B* = 1.02, *p* < 0.001). The direct effect of parental psychological control on NSSI was significant (*B* = 0.44, *p* < 0.05, OR = 1.56). The path diagram is shown in Figure 2.

The bootstrap test showed that the total indirect effect was significant (effect size = 0.89, 95% CI [0.59, 1.32]), accounting for 66.6% of the total effect. The single mediating path through self-disgust is significant (95% CI [0.28, 0.69]); the chain mediating path through self-disgust → resilience is significant (95% CI [0.04, 0.29]); and the single mediating path through resilience is significant (95% CI [0.08, 0.56]). The specific path analysis is shown in Table 5.

Parental psychological control not only has a direct positive predictive effect on NSSI but also has an effect through three indirect paths: through the mediation of self-disgust, through the mediation of resilience, and through the chain mediation of self-disgust and resilience. All indirect effects were significant according to bootstrapping.

## 4. Discussion

The results indicate that parental psychological control influences NSSI through self-disgust and resilience. Among Chinese adolescents, parental psychological control increases self-disgust, which, in turn, reduces resilience and elevates the risk of engaging in NSSI. These findings underscore the importance of focusing on parenting styles when developing family-based intervention programs aimed at preventing adolescent self-injury in China. It is essential to construct a multi-level protective system by reducing self-disgust and enhancing resilience. However, the interpretations of these findings are tempered by the study’s limitations, including its sample and cross-sectional approach. Further studies are necessary to replicate these findings in broader populations and through longitudinal designs.

Consistent with previous research and in line with Hypothesis 1, parental psychological control was a significant positive predictor of adolescents’ engagement in NSSI. The study provides evidence that parental psychological control is a contributing factor to self-injurious behaviors among Chinese adolescents, suggesting that negative parenting practices may adversely affect adolescents’ mental health. This result may be explained by several mechanisms. First, as an intrusive parenting behavior, psychological control directly exacerbates adolescents’ emotional distress by suppressing their autonomy and emotional expression, thereby increasing their risk of NSSI. Second, excessive psychological control is associated with a poorer parent–child relationship and less social support, which may contribute to NSSI indirectly through internalizing issues such as depression or anxiety. Thus, the observed predictive role of parental psychological control in adolescent NSSI may result from both direct emotional suppression and indirect deprivation of psychological resources. While this study focuses more on the pathways through which parental psychological control influences NSSI via intrapersonal factors, the role of external factors, such as social support, warrants further investigation.

Consistent with Hypotheses 2 and 3, the results of this paper indicate that parental psychological control increases Chinese adolescents’ vulnerability to non-suicidal self-injury (NSSI) by exacerbating self-disgust and reducing resilience. These results align with the existing literature and theoretical expectations. Specifically, as a form of negative parenting behavior, parental psychological control may heighten adolescents’ self-disgust by reinforcing their tendency toward self-denial and fostering biased self-attribution patterns. In the Chinese collectivist framework, where self-worth is often interdependent with family honor and social evaluation, failure to meet parental expectations can be perceived as a moral and social shortcoming. This culturally shaped attribution likely intensifies self-disgust. At the same time, it may diminish resilience by inhibiting the development of independent problem-solving abilities and impeding the acquisition of adaptive emotion regulation strategies. Together, these two mechanisms contribute to an increased risk of NSSI among adolescents. However, the specific operational mechanisms of these mediating pathways and their potential moderating factors require further investigation in future studies.

Moreover, the findings of this study support the hypothesized chain mediation model, indicating that parental psychological control indeed exacerbates adolescents’ NSSI risk by intensifying self-disgust, which, in turn, depletes psychological resilience. This discovery deepens our understanding of the processes behind the negative effects of parental psychological control, revealing a pathway from external control to internal self-aggression and eventually to the exhaustion of adaptive psychological resources. This finding can be interpreted through several mechanisms.

First, self-disgust severely erodes an individual’s positive self-perception and sense of self-worth. Individuals with high resilience typically possess positive self-schemas and have confidence in their capacity to manage their environment (X. Zhang et al., 2014). In contrast, self-disgust traps individuals in a negative self-cognitive state of “unacceptability,” hindering the belief that “I can cope with challenges” (Xi et al., 2012). At the emotional and behavioral levels, self-disgust not only consumes psychological energy and promotes the use of maladaptive strategies such as suppression and avoidance (Smith et al., 2015), leading to exacerbated emotional dysregulation, but also weakens proactive problem-solving abilities. This results in avoidance of challenges due to self-doubt and feelings of powerlessness (Xi et al., 2012), making it difficult to effectively mobilize resources to cope with difficulties. Furthermore, self-disgust impedes the acquisition and utilization of external support resources. The development of resilience relies on social support; however, individuals with high levels of self-disgust often actively distance themselves from interpersonal connections due to feelings of being “unworthy of help” or fear of rejection (Ille et al., 2014). This cuts off the external resource replenishment pathways essential for resilience, further exacerbating psychological vulnerability. These mechanisms collectively indicate that self-disgust is not merely a unidimensional emotional issue but rather a systemic process of psychological resource depletion, which provides a theoretical explanation for its role as a key mediator in this study. It should be noted that chain mediation represents a partial mediation effect, as the direct effect of parental psychological control on NSSI remained significant in this study after accounting for self-disgust and resilience. This suggests that additional mechanisms, such as emotion regulation difficulties or lack of social support, may also contribute to this relationship, and should be further explored in future research.

Therefore, the chain pathway revealed in this paper suggests that there is a vicious developmental cycle that may be activated by parental psychological control: high levels of psychological control trigger self-disgust in adolescents, which, in turn, undermines the various resources necessary for building resilience. Ultimately, this lack of resilience leads individuals to adopt NSSI as a fragile coping strategy when facing stress. This model integrates multiple factors, including family environment, self-cognition, and emotion regulation, providing a more comprehensive framework for understanding the etiology of NSSI.

Based on the above mechanisms, prevention and intervention efforts should adopt a culturally sensitive, multi-level strategy. At the family level, it is essential to educate parents on reducing psychological control while respecting the cultural importance of family harmony. This involves distinguishing autonomy-supportive care from controlling behaviors often disguised as concern, thereby promoting the adolescent’s holistic development in a manner consistent with Chinese family values. Concurrently, interventions should precisely target self-disgust as the core mediator, for example, using CBT to restructure negative self-schemas, implementing emotion regulation training to improve coping strategies, and enhancing social skills training to strengthen the ability to seek external support. Furthermore, resilience-building efforts can actively incorporate collectivist cultural assets, such as strengthening connections to supportive peers or community networks, to help mitigate the social withdrawal and negative self-perception associated with self-disgust. Together, these approaches can ultimately boost resilience and reduce the risk of NSSI.

Building on the existing literature, this study makes several distinct contributions. Firstly, at the theoretical level, this study addresses a gap in the literature by testing and supporting the chained mediation pathway from self-disgust to resilience among Chinese adolescents. This model reveals a specific internal mechanism through which parental psychological control influences NSSI, thereby advancing the understanding of its effects from general emotional distress to deeper processes of self-cognition and psychological resource depletion. Secondly, at the cultural level, this study specifically explains how, within China’s collectivist cultural context, parental psychological control exacerbates adolescents’ self-disgust through self-attribution patterns closely tied to family honor and social evaluation. This offers a culturally contextualized interpretation for the localization of related Western theories. Finally, at the practical level, the findings provide clear focal points for prevention and intervention: measures should be systematically implemented across three levels: family environment (reducing psychological control), individual cognition and emotion (targeting a reduction in self-disgust), and psychological resources (enhancing resilience). This offers an evidence-based and concrete pathway for the prevention and intervention of adolescent NSSI in the Chinese context.

There are several limitations to this study that should not be ignored, which suggest potential directions for future research. First, this study used cross-sectional data, and the direction of causal relationships between NSSI and predictive variables could not be determined. While the proposed chain mediation aligns with theoretical expectations, its applicability remains confined within a correlational framework. Future longitudinal studies are needed to implement multi-time point tracking to clarify the causal, temporal, and dynamic interactions between these variables. Second, the sample for this study consisted solely of middle school students in Chongqing, China. Consequently, the findings may not be fully generalizable to adolescents residing in rural areas, other provinces with distinct socioeconomic and cultural contexts, or other cultural settings (e.g., Western individualistic societies). Future studies should employ multi-regional or nationally representative sampling to enhance the external validity of the findings. Furthermore, future research should conduct sampling on high-risk groups potentially more vulnerable to NSSI, including dropout adolescents, rural school students, and students in technical secondary schools. Third, this study relied on self-report measures for data collection with possible response biases. Especially for sensitive topics such as parental psychological control, self-disgust, and NSSI, adolescents may underestimate or conceal the true situation due to social expectations, and their emotional state, such as depression, may affect their perception of parental behavior and the accuracy of reporting their own emotions. A multi-source assessment approach, such as structured interviews, parental reports, and clinical evaluations, is needed in future research. This approach would provide more perspective for understanding the roles that these variables play in adolescents’ NSSI behavior. Lastly, the observed partial mediation effect suggests that the model does not fully explain the pathways through which parental psychological control influences NSSI. Future studies should, thus, examine other potential mediators or moderators to develop a more comprehensive explanatory framework.

## 5. Conclusions

This study found through a cross-sectional survey of 463 middle school students that NSSI, parental psychological control, self-disgust, and resilience are significantly correlated. The chain mediation effect indicated that self-disgust and resilience sequentially mediate the relationship between parental psychological control and NSSI. These findings provide an important theoretical perspective and potential path of action for understanding the complex association between parental psychological control and adolescent NSSI. Therefore, future longitudinal or intervention studies can further examine the effectiveness of comprehensive strategies aimed at reducing parental psychological control, alleviating adolescent self-disgust, and enhancing resilience, and can effectively reduce the occurrence of NSSI. For practitioners, focusing on the links between these factors may help identify high-risk groups early and target interventions.

## Figures and Tables

**Table 1:** Correlation coefficient matrix between all variables (n = 463).

	M	SD	1	2	3	4	5	6	7
1. NSSI Score	2.55	6.06	1						
2. Parental Psychological Control	45.14	17.53	0.39 ***	1					
3. Guilt Introduction	25.76	9.78	0.39 ***	0.97 ***	1				
4. Love Withdraw	10.77	5.23	0.36 ***	0.93 ***	0.85 ***	1			
5. Authority Assertion	8.60	3.86	0.29 ***	0.83 ***	0.70 ***	0.74 ***	1		
6. Self-disgust	34.80	13.24	0.48 ***	0.50 ***	0.50 ***	0.47 ***	0.38 ***	1	
7. Resilience	90.43	17.30	−0.41 ***	−0.63 ***	−0.60 ***	−0.59 ***	−0.53 ***	−0.66 ***	1

*** *p* < 0.001.

**Table 2:** Multivariate logistic regression analysis of NSSI behavior (Crude Association).

Item	*B* (SE)	*OR* (95% CI)	*p*-Value
Parental Psychological Control	0.48	1.62 (1.13~2.33)	<0.01
Self-disgust	0.97	2.63 (1.75~3.95)	<0.01
Resilience	−0.82	0.44 (0.27~0.73)	<0.01

**Table 3:** Multivariate logistic regression analysis of NSSI behavior (Adjusted Association).

Item	*B* (SE)	*OR* (95% CI)	*p*-Value
Parental Psychological Control	0.45	1.58 (1.08~2.30)	<0.05
Self-Disgust	1.00	2.73 (1.78~4.18)	<0.01
Resilience	−0.82	0.44 (0.26~0.75)	<0.01
Gender			
Female	Ref		
Male	−0.66	0.52 (0.26~1.02)	0.59
Grade			
Grade 11	Ref		
Grade 7	−0.38	0.68 (1.57~2.99)	0.61
Grade 8	1.12	3.06 (0.47~20.05)	0.24
Family Type			
Other Family Type	Ref		
Two-parent Family	−2.20	0.11 (0.02~6.25)	0.29
Single-parent Family	−2.29	0.10 (0.02~6.23)	0.28
Stepfamily	−3.17	0.04 (0.00~3.02)	0.15
Only-Child Status			
No	Ref		
Yes	0.25	1.28 (0.58~2.42)	0.45

**Table 4:** Path analysis of parental psychological control on NSSI.

Path	*B*	*SE*	*p*-Value	95% CI	*OR*
Parental Psychological Control → Self-Disgust	0.44	0.04	<0.001	[0.36, 0.52]	-
Self-Disgust → Resilience	−0.43	0.04	<0.001	[−0.51, −0.36]	-
Parental Psychological Control → Resilience	−0.37	0.04	<0.001	[−0.45, −0.30]	-
Self-disgust → NSSI	1.02	0.22	<0.001	[0.60, 1.45]	-
Resilience → NSSI	−0.78	0.27	<0.001	[−1.30, −0.25]	0.46
Parental Psychological Control → NSSI	0.44	0.19	<0.05	[0.07, 0.81]	1.56

**Table 5:** Indirect effects analysis of self-disgust and resilience (bootstrap = 5000).

Path	Indirect Effect	Boot SE	Boot LLCI	Boot ULCI
Total Indirect Effect	0.89	0.19	0.59	1.32
Path 1: Parental Psychological Control → Self-Disgust → NSSI	0.45	0.11	0.28	0.69
Path 2: Parental Psychological Control → Resilience → NSSI	0.29	0.12	0.08	0.56
Path 3: Parental Psychological Control → Self-Disgust → Resilience → NSSI	0.15	0.06	0.04	0.29

## Data Availability

The data used in the study are available upon request to the corresponding author.
